# CT三维容积分析在实性肺结节恶性风险度评估中的价值

**DOI:** 10.3779/j.issn.1009-3419.2016.05.05

**Published:** 2016-05-20

**Authors:** 梦琦 李, 融城 韩, 文静 宋, 欣悦 王, 芳芳 郭, 大同 苏, 铁链 于, 颖 王

**Affiliations:** 1 300052 天津，天津医科大学总医院医学影像科 Department of Radiology, Tianjin Medical University General Hospital, Tianjin 300052, China; 2 300052 天津，天津医科大学总医院医学病理科 Department of Pathology, Tianjin Medical University General Hospital, Tianjin 300052, China

**Keywords:** 肺结节, 计算机体层摄影, 容积, 三维形态分析, 风险度, Pulmonary nodules, Computed tomography, Volume, Three-dimensional volumetric analysis, Cancer risk assessment

## Abstract

**背景与目的:**

肺结节临床处理策略主要基于其恶性风险度评估，目前公认的计算机断层扫描（computed tomography, CT）影像学标准为结节直径。容积CT及三维分析软件的应用使肺结节形态学特征显示更加清晰。本研究的目的是评估肺结节的容积及三维形态特征（边缘、形状、位置）在结节恶性风险度评估中的价值。

**方法:**

应用三维分析软件对200例直径小于3 cm实性结节的CT影像资料进行回顾性分析，恶性结节经病理或组织学确认，良性结节经病理或两年随访无增大确认。对全部结节及亚厘米结节（直径小于10 mm）分别采用*Logistic*回归分析计算结节三维边缘（光滑、分叶、毛刺或不规则）、形状（球体、非球体）、位置（肺实质内、血管相贴、胸膜相贴）、结节容积的似然比（odds ratios, ORs），并通过受试者工作特征（receiver operating characteristic, ROC）曲线确定结节容积评估恶性风险度的最佳阈值。

**结果:**

纳入研究的200例中恶性78例，良性122例。对全部结节的*Logistic*回归分析结果显示结节容积（OR=3.3, *P* < 0.001）、边缘形态（分叶、毛刺或不规则的OR值分别为13.4、9.8，*P*均=0.001）具有预测价值，而结节的位置及三维形状不具备预测价值（*P*>0.05）。ROC分析显示结节容积对恶性风险度评估有价值（曲线下面积为0.928；*P* < 0.01，最佳阈值为666 mm^3^）。对亚厘米结节的分析显示仅边缘分叶、毛刺或不规则的结节恶性风险度高（OR=60.5, 75.0; *P*=0.003, 0.007）。

**结论:**

结节的三维容积及边缘形态有助于判定结节的恶性风险度，容积大于666 mm^3^可作为恶性高危结节的阈值；边缘分叶、毛刺或不规则是亚厘米结节的唯一高危因素。

## Abstract (en)

**Background and objective:**

The management of pulmonary nodules relies on cancer risk assessment, in which the only widely accepted criterion is diameter. The development of volumetric computed tomography (CT) and three-dimensional (3D) software enhances the clarity in displaying the nodules' characteristics. This study evaluated the values of the nodules' volume and 3D morphological characteristics (edge, shape and location) in cancer risk assessment.

**Methods:**

The CT data of 200 pulmonary nodules were retrospectively evaluated using 3D volumetric software. The malignancy or benignity of all the nodules was confirmed by pathology, histology or follow up (>2 years). *Logistic* regression analysis was performed to calculate the odds ratios (ORs) of the 3D margin (smooth, lobulated or spiculated/irregular), shape (spherical or non-spherical), location (purely intraparenchymal, juxtavascular or pleural-attached), and nodule volume in cancer risk assessment for total and sub-centimeter nodules. The receiver operating characteristic (ROC) curve was employed to determine the optimal threshold for the nodule volume.

**Results:**

Out of 200 pulmonary nodules, 78 were malignant, whereas 122 were benign. The *Logistic* regression analysis showed that the volume (OR=3.3; *P* < 0.001) and the 3D margin (OR=13.4, 9.8; both *P*=0.001) were independent predictive factors of malignancy, whereas the location and 3D shape exhibited no total predictive value (*P*>0.05). ROC analysis showed that the optimal threshold for malignancy was 666 mm^3^. For sub-centimeter nodules, the 3D margin was the only valuable predictive factor of malignancy (OR=60.5, 75.0; *P*=0.003, 0.007).

**Conclusion:**

The volume and 3D margin are important factors considered to assess the cancer risk of pulmonary nodules. Volumes larger than 666 mm^3^ can be determined as high risk for pulmonary nodules; by contrast, nodules with lobulated, spiculated, or irregular margin present a high malignancy probability.

肺癌死亡率在所有恶性肿瘤中居首位^[1]^。我国肺癌发病年增长率约26.9%，预计2025年患病人数将高达100万^[2]^。肺癌患者的生存率和肿瘤分期密切相关，早期肺癌（T1a期）的5年生存率高达77%，而晚期肺癌（M1）的5年生存率只有3%^[3]^。肺结节是肺癌的常见计算机断层扫描（computed tomography, CT）表现，对偶然发现的肺结节恶性风险度进行评估是决定采取何种处理策略的关键。

目前肺结节影像特征的恶性风险度评估研究主要基于二维图像，其潜在缺陷在于二维图像提供的信息不完整^[4]^，而三维（3D）容积分析技术能够更客观的反映出结节整体的容积及其形态特征^[4]^。因此，本研究的目的是应用肺结节三维容积分析软件对肺结节进行分割，测量结节的容积，观察结节的三维形状，评估其在判定肺内结节风险度方面的价值，尤其是亚厘米肺结节，进而为肺结节的处理策略提供依据。

## 1 材料与方法

### 1.1 患者资料

回顾性分析2010年9月-2015年6月期间在天津医科大学总医院放射科行GE 16排或64排螺旋CT检查发现肺结节病例。①纳入标准：行手术或穿刺获得病理的肺内实性结节；偶然发现肺内实性结节并连续随访两年及以上；肺结节直径均小于3 cm。②排除标准：具有明显感染症状及肺内炎性CT表现。恶性结节经病理或组织学确认，良性结节经病理或两年随访无增大（体积变化小于25%）确认。手术或穿刺获得病理患者共84例，男性44例，女性40例，平均年龄（61±8.6）岁，共纳入88个肺结节（恶性78例、良性10例）。连续随访两年及以上的患者共79例，男性45例，女性34例，平均年龄（64±10.5）岁，共纳入112个结节，随访时间范围为740天-2, 223天，平均为（1, 404±502）天。

### 1.2 图像采集

选取所选病例首次CT图像进行分析。所有患者均在GE 16排或64排螺旋CT机进行扫描，扫描范围自胸廓入口至肺底部。所有扫描均在患者一次吸气并屏气后完成全肺扫描，扫描方式为螺旋扫描，电压120 KV，电流300 mA，螺距1.375:1，扫描层厚5 mm，机架螺旋一周时间0.4 s，显示野（field of view, FOV）360 mm，图像矩阵512×512，重建算法为默认标准算法，重建1.25 mm层厚轴位图像。

### 1.3 图像分析

三维结节测量均在GEAW4.6工作站进行，分别由两位放射科医生（诊断经验分别为2年、5年）独立对结节进行分析，判断结节的位置及三维容积形态。两者意见不一致者提交给第三位诊断医生（诊断经验20年）进行决断。结节的位置分为三种：肺实质内、血管相贴以及胸膜相贴。应用ALA（advanced lung analysis, GE）自动分析软件对肺结节进行三维容积分割，具体操作步骤：选择1.25 mm层厚图像，进入ALA程序界面后，在打开的轴位图像上由观察者用鼠标点击需要分析结节的中心，软件即自动对结节进行分割并显示出分割后结节的三维容积及轴位分割图像，对于分割不满意的结节需要进行手动调整，通过调整鼠标的选取点、设置结节轴位的提取范围、并可以根据结节的位置的不同选取不同的重建算法来达到最佳的效果，取两位放射医生的平均值作为结节的容积。结节的形态学特点评估均基于三维容积图像进行分析，目的是发现不同截面上结节的形态特点。结节的三维边缘形态分为三种：光滑、分叶、毛刺或不规则；三维形状分为两种：球体及非球体。结节的二维径线测量在1.25 mm层厚标准算法的横断面图像上选取结节最大层面，应用电子卡尺测量其最大径（X）及相应垂直径（Y），并用1.25 mm层厚图像重建矢状位及冠状位，测量结节最大头尾径（Z），取三者平均值作为结节的直径。

### 1.4 统计学方法

以结节性质作为因变量，结节容积（因为结节容积不符合正态分布，故首先进行自然对数转换-lnV）、结节三维形状（球体、非球体），三维边缘形态（光滑、分叶、毛刺或不规则）、结节位置（完全肺实质内，与血管相贴，与胸膜相贴）作为自变量，首先采用单因素分析确定可纳入多因素分析的变量，其次使用多因素*Logistic*回归判定各个影像学特性的恶性风险度似然比（odd ratio, OR）。由于在临床实践中，亚厘米结节定性困难，正电子发射型计算机断层显像（positron emission tomography-CT, PET-CT）、增强检查、穿刺活检等检查效能不佳，因此我们对10 mm以下结节单独进行了*Logistic*回归分析。应用受试者工作曲线评估结节容积对恶性结节的预测价值。所有统计分析均采用SPSS 17.0统计分析软件完成，以*P* < 0.05为差异有统计学意义。

## 2 结果

### 2.1 病理

两组研究对象共发现结节200例：其中经手术证实恶性结节共78例，结节平均直径（17.1±5.8）mm，其中腺癌65例、小细胞肺癌7例、细支气管肺泡癌3例、鳞状细胞癌2例、大细胞神经内分泌癌1例；手术证实良性结节10例，慢性炎症-纤维组织2例、结核球3例、错构瘤3例、淀粉样瘤1例、淋巴组织1例；连续随访两年以上结节无增大112例。

### 2.2 结节影像学特征

在所有结节中，肺实质内90例、贴血管42例、贴胸膜68例；三维边缘光滑112例、分叶27例、毛刺或不规则61例；三维形态为球体101例、非球体99例（表 1）。

**1:** 200例实性结节的影像学特征及单因素*Logistic*回归分析 The imaging features and the results of univariate *Logistic* regression analysis of 200 solid pulmonary nodules

Item	Benign (*n*=122)	Malignant (*n*=78)	OR (95%CI)	*P*
Location				
Purely intraparenchymal	69 (76.7%)	21 (23.3%)	1	
Juxtavascular	18 (42.9%)	24 (57.1%)	4.4 (2–9.6)	< 0.001
Pleural-attached	35 (51.5%)	33 (48.5%)	17.7 (1.6-6.1)	< 0.001
3D shape				
Spherical	84 (83.2%)	17 (16.8%)	1	
Non-spherical	38 (38.4%)	61 (61.6%)	7.9 (4.1-15.3)	< 0.001
Morphology				
Smooth	101 (90.2%)	11 (9.8%)		
Lobulated	7 (25.9%)	20 (74.1%)	26.2 (9.1-75.9)	< 0.001
Spiculated/Irregular	14 (23.0%)	47 (77.0%)	30.8 (13.0-73.0)	< 0.001
lnV			4.4 (3.1-6.2)	< 0.001
Mean diameter (mm)				
0 < d≤10	105 (93.8%)	7 (6.3%)		
10 < d≤30	17 (19.3%)	71 (80.7%)		
Mean volume (mm^3^)				
0 < V≤100	48 (98.0%)	1 (2.0%)		
100 < V≤300	46 (88.5%)	6 (11.5%)		
300 < V≤500	16 (94.1%)	1 (5.9%)		
> 500 mm^3^	12 (14.6%)	70 (85.4%)		
lnV: nature logarithm of the volume.				

### 2.3 实性结节恶性风险度评估

单因素*Logistic*分析显示结节三维形状、边缘、结节位置及容积均对预测恶性结节有意义（所有*P* < 0.001），分叶、毛刺或不规则、与血管或胸膜相贴、非球体及较大的容积较边缘光滑、肺实质内、球体及较小容积结节具有更高的恶性风险度。将上述变量纳入多因素*Logsitic*回归分析后，结果显示仅结节的容积及结节的三维边缘具有预测意义，分叶结节（*P*=0.001）、毛刺或不规则（*P*=0.001）、结节容积（*P* < 0.001）的恶性风险度高（表 2）。受试者工作特征曲线（receiver operating characteristic curve，ROC曲线）分析结果显示以结节容积作为预测恶性因素，曲线下面积（area under curve, AUC）=0.928（*P* < 0.001），判断恶性的最佳阈值为666 mm^3^，相对应的灵敏度为0.89，特异度为0.93（图 1）。对于直径 < 10 mm结节：结节边缘分叶、毛刺或不规则对结节的恶性风险度评估具有统计学意义（*P*=0.003、0.007），而结节的容积不具备预测价值（*P*=0.44）（表 3）。

**2:** 实性肺结节恶性风险度多因素*Logistic*回归分析 The cancer risk assessment of 200 solid pulmonary nodules by multi-variate *Logistic* regression analysis

Item	*n*	OR	95%CI	*P*
Location				
Purely intraparenchymal	90	1		
Juxtavascular	42	2.0	0.5-7.3	0.317
Pleural-attached	68	1.6	0.5-5.7	0.449
Morphology				
Smooth	112	1		
Lobulated	27	13.4	2.9-61.8	0.001
Spiculated/Irregular	61	9.8	2.4-39.5	0.001
3D shape				
Spherical	101	1		
Non-spherical	99	1.0	0.3-3.7	0.984
lnV	200	3.3	2.3-4.8	< 0.001

**1:**
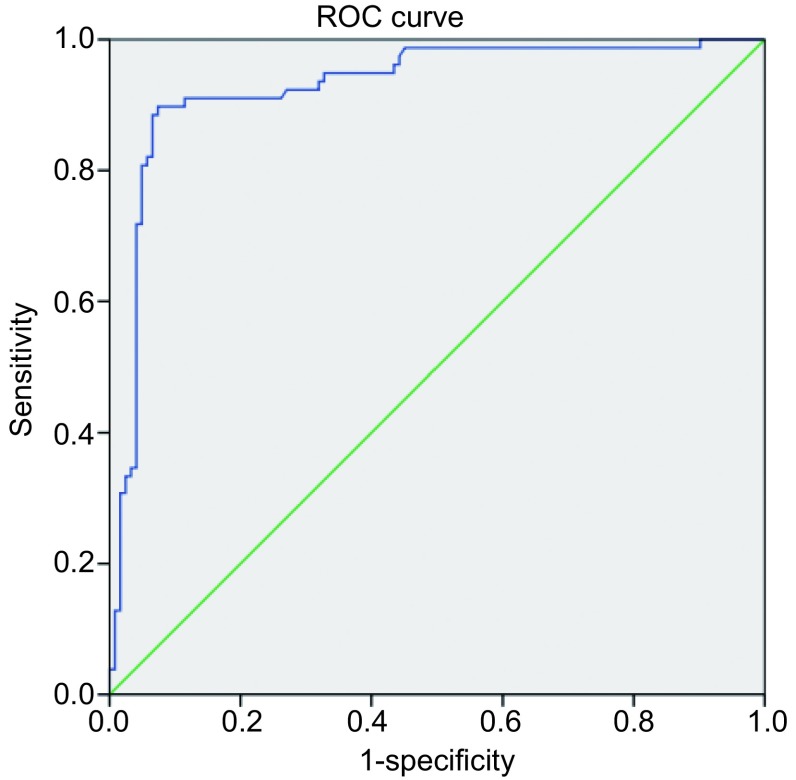
实性结节容积评估恶性危险度的ROC曲线 The ROC curve of volume to assess the cancer risk. ROC: receiver operating characteristic curve

**3:** 112例亚厘米结节恶性危险度多因素*Logistic*回归分析 The cancer risk assessment of 200 solid pulmonary nodules by multi-variate *Logistic* regression analysis

Nodule characteristics	*n*	OR	95%CI	*P*
Location				
Purely intraparenchymal	64	1		0. 91
Juxtavascular	16	1.1	0.09-14.4	0.93
Pleural-attached	32	0.6	0.02-16.5	0.78
Morphology				
Smooth	92	1		0.002
Lobulated	6	60.5	4.1-887.5	0.003
Spiculated/Irregular	14	75.0	3.3-1, 685.8	0.007
3D Shape				
Spherical	78	1		
Non-spherical	34	16.1	0.7-350.5	0.08
lnV	112	1.8	0.4-7.4	0.44

## 3 讨论

对偶然发现的肺内小结节恶性风险度进行评估具有重要的临床意义，一方面实现恶性结节的早期发现和治疗，另一方面避免对良性结节进行不必要的手术。确定肺结节的恶性风险度是制定肺结节治疗策略的关键。我们的研究发现结节的容积、边缘形态特征对评估恶性风险度均具有价值。结节容积大于666 mm^3^可以作为高危恶性结节的阈值。对于小于10 mm的小结节，边缘为分叶、毛刺或不规则的肺结节恶性风险度高。

结节容积是恶性结节的独立预测指标。在我们研究中，容积在0 mm^3^-100 mm^3^之间的结节恶性比例为2.0%，100 mm^3^-300 mm^3^为11.5%，300 mm^3^-500 mm^3^为5.9%，>500 mm^3^为85.4%。在早期肺癌筛查计划（Early Lung Cancer Action Project, ELCAP）中，小于5 mm的结节的恶性病例为1%，6 mm-10 mm为24%，11 mm-20 mm为33%，大于20 mm为80%^[5]^。Wahidi^[6]^的研究中结节的直径5 mm以下、6 mm-10 mm、10 mm-20 mm和>20 mm的恶性病例分别为1%、6%-28%、33%-64%和64%-82%。NELSON筛查试验的7, 155例受试者共发现9, 681例非钙化结节，容积 < 100 mm^3^的结节恶性病例为0.6%，100 mm^3^-300 mm^3^之间为2.4%，>300 mm^3^为16.9%^[7]^。结节恶性比例在不同研究中存在差异，其主要原因为目标人群的选择偏倚，例如大规模人群筛查研究与临床研究的病例样本所代表的总体人群具有明显不同^[8]^，然而，其总体趋势是一致的，即随着结节增大，其恶性病例也相应提高。鉴于结节的容积对判断结节恶性风险度有重要价值，我们进一步确定具有最高诊断价值的容积阈值，期望以此作为临床决策分层的依据。ROC分析得出的容积阈值为666 mm^3^，相当于直径为11 mm的球体，其对应的灵敏度为0.89，特异度为0.93。即对于肺内的结节，当容积>666 mm^3^时高度怀疑是恶性的肺结节。其结果和目前肺癌筛查常用的结节大小标准（直径>10 mm或容积>500 mm3）基本一致。

在临床实践中，对于直径大于10 mm的结节，应用PET-CT、CT强化、穿刺活检等检查方法常可明确诊断，而这些检查方法对亚厘米结节的诊断效能不佳。因此，如何对亚厘米结节进行恶性风险评估为临床亟待解决的问题。我们对亚厘米结节的回归分析显示结节的三维边缘形态为分叶、毛刺或不规则是独立的恶性评估指标，而容积对其良恶性评估无明显价值（图 2，图 3）。我们分析原因可能在于亚厘米良性结节生长缓慢，对周围结构的影响较小，边界一般会规则、光滑，出现分叶和毛刺的概率低^[9]^，而恶性结节由于癌细胞向各个方向的生长速度不对称容易形成分叶，其对周围结构的牵拉或癌细胞沿周围的小叶间隔、血管、淋巴管及细支气管向周围浸润会导致毛刺的形成。Xu等^[10]^对5 mm-10 mm的肺内非钙化结节的研究显示，边界光滑的结节随访一年后，恶性发生率为0，而对于边界不光滑的结节来说，结节的恶性度主要依据的是结节的直径，而结节的形态与良恶性关系不明显。我们的研究与其存在一定的差别。其原因可能在于我们的结节选择中将具有明确急性感染症状病例排除。根据我们的经验，感染性结节出现不光滑边缘的比例较高，与恶性结节具备一定的重叠，但在临床实践中，结合病例的临床信息及短期抗炎治疗常可鉴别。其次在于样本选择的不同，前者为筛查病例，我们为临床肺结节病例，肺癌的阳性率不同。我们研究结果的意义在于确定边缘分叶、毛刺或不规则为亚厘米结节的恶性高危指标，需要引起临床的高度警惕，短期密切随访或手术切除为可行的选择。对亚厘米结节，我们发现结节容积的预测价值不明显，可能与样本中300 mm^3^-500 mm^3^之间的结节数目较少有关，因此该结论尚有待更大样本量的研究确定。

**2:**
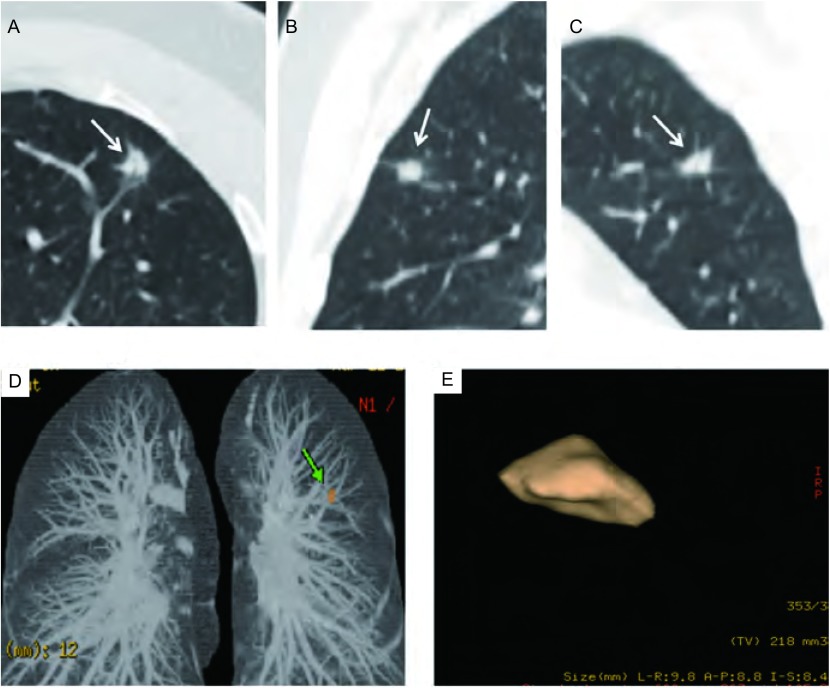
男性，47岁，A-D：左肺上叶实性结节，边缘形态不规则，呈分叶状，手动测得三维最大径线分别为8.7 mm、7.4 mm及7.6 mm；E：三维自动容积分析，结节为非球形，自动容积分析结节容积为218 mm^3^，自动测得最大径分别为9.8 mm、8.8 mm、8.4 mm，病理结果为浸润性腺癌 Male, 47 years old, A-D: a nodule (white arrow) was incidentally detected in left upper lobe, nodules margin is lobulated, manually measured the largest diameter in X, Y and Z was 8.7 mm, 7.4 mm, 7.6 mm respectively; E:3D volumetric analysis showed nodule was non-spherical, software-generated volume of the nodule was 218 mm^3^. Automatic measured the maximum diameter was 9.8 mm, 8.8 mm, 8.4 mm respectively. Pathology result was invasive adenocarcinoma

**3:**
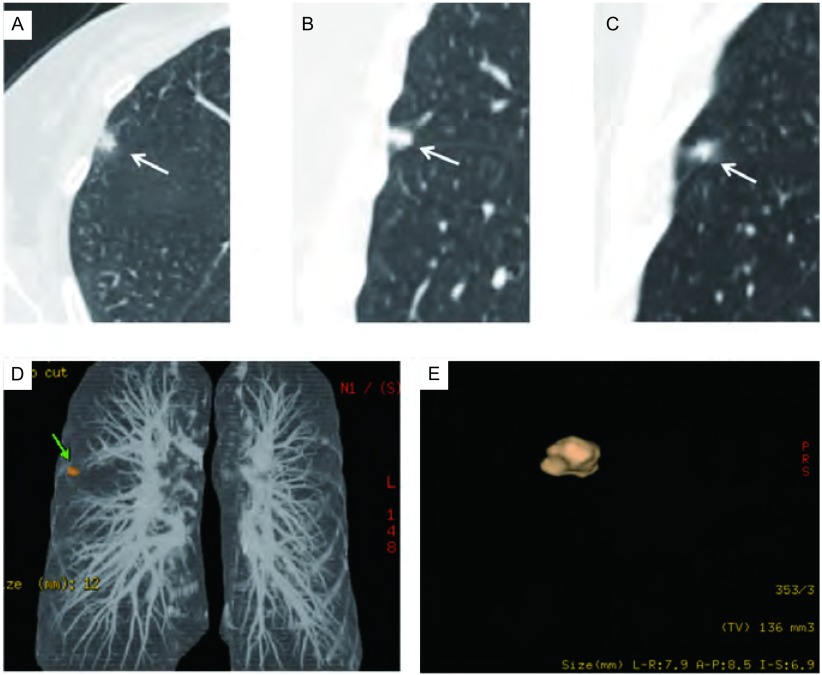
女性，53岁，A-D：右肺上叶实性结节，边缘不规则，手动测得三维最大径线分别为6.0 mm、5.7 mm及5.9 mm。E：三维自动容积分析，结节为非球形，自动容积分析结节容积为136 mm^3^，自动测得最大径分别为7.9 mm、8.5 mm、6.9 mm，病理结果为腺癌 Female, 53 years old, A-D: a nodule (white arrow) was incidentally detected in right upper lobe, nodules margin is irregular, manually measured the largest diameter in X, Y and Z was 6.0 mm, 5.7 mm, 5.9 mm respectively; E: 3D volumetric analysis showed nodule was non-spherical, software-generated volume of the nodule was 136 mm^3^. Automatic measured the maximum diameter was 7.9 mm, 8.5 mm, 6.9 mm respectively. Pathology result was adenocarcinoma

我们研究中的所有结节分析均基于CT三维容积分析技术。与常规二维评价相比，结节的容积定量较二维直径测量更准确、重复性更好，更适合作为决策分层的标准。其次，三维容积图像相对于二维图像更能精确反映结节整体的边缘形态。在二维轴位图像上结节可能是光滑的，但在冠状位或矢状位图像上可表现为分叶或毛刺，使得整个结节的形态不规则。在临床实践中，我们推荐基于三维容积分析的结节边缘特征评估。

我们研究存在一定的局限性。首先，由于临床实践中肺结节随访患者失访率较高，故采用的是回顾性研究，和前瞻性队列研究相比诊断效能不足。其次，亚厘米结节的恶性病例数相对较少。

总之，肺结节的三维容积分析对评估结节恶性风险度具有价值，容积大于666 mm^3^可以作为高危结节的阈值，对于亚厘米结节，边缘分叶、毛刺及不规则是恶性的高危因素。
